# Predicting the risk of falling – efficacy of a risk assessment tool compared to nurses' judgement: a cluster-randomised controlled trial [ISRCTN37794278]

**DOI:** 10.1186/1471-2318-5-14

**Published:** 2005-11-10

**Authors:** Gabriele Meyer, Sascha Köpke, Ralf Bender, Ingrid Mühlhauser

**Affiliations:** 1 University of Hamburg, Unit of Health Sciences and Education, Martin-Luther-King-Platz 6, D-20146 Hamburg, Germany; 2 Institute for Quality and Economic Efficiency in Health Care, Dillenburger Straße 27, D-51105 Köln, Germany

## Abstract

**Background:**

Older people living in nursing homes are at high risk of falling because of their general frailty and multiple pathologies. Prediction of falls might lead to an efficient allocation of preventive measures. Although several tools to assess the risk of falling have been developed, their impact on clinically relevant endpoints has never been investigated. The present study will evaluate the clinical efficacy and consequences of different fall risk assessment strategies.

**Study design:**

Cluster-randomised controlled trial with nursing home clusters randomised either to the use of a standard fall risk assessment tool alongside nurses' clinical judgement or to nurses' clinical judgement alone. Standard care of all clusters will be optimised by structured education on best evidence strategies to prevent falls and fall related injuries. 54 nursing home clusters including 1,080 residents will be recruited. Residents must be ≥ 70 years, not bedridden, and living in the nursing home for more than three months. The primary endpoint is the number of participants with at least one fall at 12 months. Secondary outcome measures are the number of falls, clinical consequences including side effects of the two risk assessment strategies. Other measures are fall related injuries, hospital admissions and consultations with a physician, and costs.

## Background

Fall prevention in the elderly has been recognised as an important area of research and intervention [1]. Numerous studies have presented combinations of fall related risk factors and a number of risk assessment tools have been developed [2,3]. However, few of these tools are based on rigorous research. The minority has been adequately tested for accuracy [4]. Reproducibility and transportability have been rarely investigated [5]. An own recently conducted systematic review including 27 publications on 25 non-laboratory fall risk assessment tools found that only 13 instruments have been validated in different settings [6]. Treatment paradox has been discussed by only four publications although it seems to be an important source of bias in accuracy studies which use the number of fallers as reference standard. However, there is no other measure to use as the gold standard for determining the validity of a fall risk assessment tool. Treatment paradox is difficult to overcome as it would be unethical to discourage fall prevention measures in the clinical setting in order to test risk assessment tools. None of the publications included in the systematic review [6] reported or even discussed side effects of fall risk assessment like the application of physical restraints.

A German national nursing guideline on fall prevention discourages the use of a risk assessment tool [7]. However, as shown in a national survey these instruments are increasingly used throughout different nursing settings [8].

Only three tools have been repeatedly evaluated in geriatric populations: the Tinetti Test, the Mobility Interaction Fall Chart (MIF) and the Downton Index [9-11]. The Tinetti Test [9] and the MIF [10] are not suitable for routine nursing assessment in nursing homes as they are time-consuming and require special training whereas the Downton Index has been described to be easily administered by nurses [11]. Few accuracy studies compared fall risk assessment tools to nurses' clinical judgement [4,10,12,13]. Predictive values were unsatisfactory for both risk assessment strategies. None of the tools was superior to nurses' clinical judgement.

The impact of the use of a fall risk assessment tool on clinically relevant endpoints has never been investigated within a methodologically rigorous trial. Therefore, we designed a randomised controlled trial to compare the clinical efficacy and consequences of the use of a standard fall risk assessment tool alongside nurses' clinical judgement with nurses' clinical judgement alone. We chose the Downton Index as comparator to single nurses' clinical judgement since it has been validated in a nursing home population and its predictive value is comparable to other instruments. The Downton Index is easy to administer and is comparable to those non-validated tools already in use in German nursing homes.

## Methods

### Study design and setting

The study is a cluster-randomised controlled trial over 12 months with nursing homes randomised either to optimised standard care and the use of the Downton Index or optimised standard care alone. A full economic evaluation is also being conducted.

### Ethical considerations

The protocol has been approved by the ethics committee of the Hamburg chamber of physicians and the regional data protection office.

### Study interventions

A structured single education session of 60 to 90 minutes will be provided for all clusters to optimise standard care and to minimise possible centre effects. The education programme will cover information on best evidence strategies to prevent falls and fall related injuries. The programme is based on principles of social learning theory [14]. The development of the curriculum followed approaches we have successfully tested for other education programmes [15,16].

After randomisation nurses of the intervention group (IG) will be introduced to the use of the Downton Index. A nominated nurse in charge of each cluster will then be responsible for the monthly application of the tool. No further intervention will be carried out in the control group.

### Identification of clusters and participants

Nursing homes in the cities Hamburg and possibly Bremen, Germany, and respective catchment areas will be invited to participate. A cluster is defined as a nursing home by itself or an independently working ward of a large nursing home. In a first step, a serially numbered list of all residents of each cluster will be produced by the nurse in charge. In a second step 20 residents fulfilling the predefined inclusion criteria (≥ 70 years old, not bedridden, and living in the nursing home for more than three months) will be selected using investigators' random table. Descriptive data on the cluster and participating residents will be collected by the nurse in charge supported by the external investigators. The figure shows the summary of the trial design.

### Randomisation

We will use computer generated randomisation lists for concealed allocation of clusters. To obviate disparate sample sizes permuted blocks will be used. Clusters will be allocated by an external researcher immediately after collection of baseline data and administration of the education programme.

### Clinical outcomes measures

The primary outcome is the number of participants with at least one fall at 12 months. Secondary outcome measures are the number of falls, clinical consequences, i.e. fall and injury prevention measures applied, and side effects of the two risk assessment strategies. Side effects are defined as the application of physical or pharmacological restraints. Injuries and fractures related to falls, hospital admissions and consultations with a physician related to falls irrespective of the reason for falls, and costs will also be recorded.

Nursing staff will have to fill in a specially developed documentation sheet in case of a fall event, also a documentation sheet on measures used to prevent falls once a month.

Data will be checked monthly during personal visits of the investigators. It is not possible to objectify the documented falls. Nevertheless, in Germany, nurses are legally required to document falls in nursing homes. Interrater reliability of the Downton Index will be determined in a subgroup of nurses of the IG clusters.

### Sample size calculation

It is assumed that about 45% of the participants in the control group will experience at least one fall in 12 months with an intra-class correlation coefficient of ICCC = 0.075. A cluster-randomisation with about 20 participants in each cluster leads to a variation inflation factor of VIF = 2.425. Assuming that 20% of the participants will not complete follow-up and furthermore assuming an absolute difference of 15% (incidence of fallers in the Downton Index-group: 30%) to reach a significant result to the level of alpha = 0.05 with a power of 80% a total sample size of n = 1,080 participants is needed. Therefore the sample size comprises a total of 54 clusters with about 20 participants each.

### Statistical analysis

The main outcome is the proportion of persons with at least one fall and will be analysed by using a chi-square test adjusted for cluster randomisation [17]. The effect of the risk assessment tool will be expressed as relative risk, difference in absolute risk, and number needed to treat. For risk differences 95% confidence intervals will be calculated using a method appropriate for cluster-randomised trials [18]. For all other follow up data the cluster will be used as unit of analysis. For statistical comparisons between the groups the Wilcoxon rank sum test will be performed. Two-sided p ≤ 0.05 will be regarded as significant. Interrater reliability will be calculated using kappa statistics.

### Economic evaluation

The economic evaluation will adopt the viewpoint of the German health and nursing care insurance, adding up all costs and savings relevant from the viewpoint of health care insurers and party payers. Assessment will include costs for optimisation of usual care for both groups, costs for using the fall risk assessment tool in the IG as well as medical and nursing care costs following falls for both groups. Costs for the interventions will be estimated based on information from trial records. The analysis will adopt an incremental approach such that data collection will concentrate on resource use differences between study groups. The process of collecting data on resource use will be undertaken separately from data collection on unit costs. Resource use data due to fall related health care will be collected by the investigators during the personal visits using a cost component protocol. The documentation sheet has been successfully evaluated in a recent economic evaluation of a randomised controlled trial [19]. Unit costs will be collected from different sources including published data, health insurances, and various health care providers.

At present, it is not possible to state with certainty which form of economic analysis will be employed, since this will be driven in part by the clinical study results. If a difference in the primary endpoint is observed, then a cost efficacy analysis will be conducted. Otherwise a cost comparison analysis will be conducted. Since cost data will be available for the duration of the trial only, the appropriate effectiveness measure is the one that allows treatment effects during the trial only. A sensitivity analysis on the key variables that might influence the result of the economic evaluation will be carried out.

### Time plan

The pilot phase of the study started in September 2005. Consecutive recruitment of clusters has started at the same time and is expected to last 4 to 6 months.

## Competing interests

The author(s) declare that they have no competing interests.

## Authors' contributions

Gabriele Meyer, Sascha Köpke and Ingrid Mühlhauser were responsible for identifying the research question, and equally contributed to the development of the protocol and study design. Gabriele Meyer and Sascha Köpke were responsible for the drafting of this paper, Ingrid Mühlhauser commented on the drafts and approved the final version. Ralf Bender contributed as statistician.

**Figure 1:**
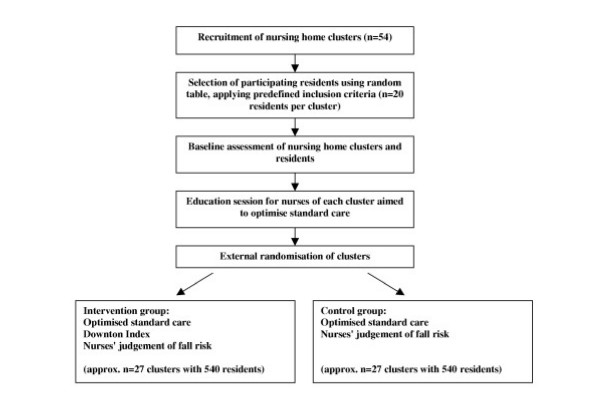
Summary of trial design.

## Pre-publication history

The pre-publication history for this paper can be accessed here:



## References

[B1] Gillespie LD, Gillespie WJ, Robertson MC, Lamb SE, Cumming RG, Rowe BH (2003). Interventions for preventing falls in elderly people. Cochrane Database Syst Rev.

[B2] Perell KL, Nelson A, Goldman RL, Luther SL, Prieto-Lewis N, Rubenstein LZ (2001). Fall risk assessment measures: an analytic review. J Gerontol A Biol Sci Med Sci.

[B3] WHO What are the main risk factors for falls amongst older people and what are the most effective interventions to prevent these falls? How should interventions to prevent falls be implemented?. http://www.euro.who.int/document/E82552.pdf.

[B4] Myers H, Nikoletti S (2003). Fall risk assessment: a prospective investigation of nurses' clinical judgement and risk assessment tools in predicting patient falls. Int J Nurs Pract.

[B5] Justice AC, Covinsky KE, Berlin JA (1999). Assessing the generalizability of prognostic information. Ann Intern Med.

[B6] Köpke S, Lange H, Meyer G (2004). Validität von Tests zur Einschätzung des Sturzrisikos älterer Menschen [Validity of instruments to predict the risk of falling in the elderly] [abstract]. Z Gerontol Geriatr.

[B7] Deutsches Netzwerk für Qualitätsentwicklung in der Pflege Expertenstandard Sturzprophylaxe. http://www.dnqp.de/ExpertenstandardSturz.pdf.

[B8] Dassen T Prävalenz: Pflegeabhängigkeit, Sturzereignisse, Inkontinenz, Dekubitus, Erhebung 2003. Charité, Universitätsmedizin Berlin, Zentrum für Human- und Gesundheitswissenschaft, Institut für Medizin / Pflegepädagogik und Pflegewissenschaft.

[B9] Raîche M, Hebert R, Prince F, Corriveau H (2000). Screening older adults at risk of falling with the Tinetti balance scale. Lancet.

[B10] Lundin-Olsson L, Jensen J, Nyberg L, Gustafson Y (2003). Predicting falls in residential care by a risk assessment tool, staff judgement, and history of falls. Aging Clin Exp Res.

[B11] Rosendahl E, Lundin-Olsson L, Kallin K, Jensen J, Gustafson Y, Nyberg L (2003). Prediction of falls among older people in residential care facilities by the Downton index. Aging Clin Exp Res.

[B12] Moore T, Martin J, Stonehouse J (1996). Predicting falls: risk assessment tool versus clinical judgement. Perspectives.

[B13] Eagle DJ, Salama S, Whitman D, Evans LA, Ho E, Olde J (1999). Comparison of three instruments in predicting accidental falls in selected inpatients in a general teaching hospital. J Gerontol Nurs.

[B14] Bandura A (1977). Social learning theory.

[B15] Mühlhauser I (1993). Verbesserung der Behandlungsqualität der chronischen Krankheiten Diabetes mellitus, arterielle Hypertonie und Asthma bronchiale durch strukturierte Therapie- und Schulungsprogramme.

[B16] Meyer G, Warnke A, Bender R, Muhlhauser I (2003). Effect on hip fractures of increased use of hip protectors in nursing homes: cluster randomised controlled trial. Br Med J.

[B17] Donner A, Klar N (1994). Methods for comparing event rates in intervention studies when the unit of allocation is a cluster. Am J Epidemiol.

[B18] Donner A, Klar N (1993). Confidence interval construction for effect measures arising from cluster randomization trials. J Clin Epidemiol.

[B19] Meyer G, Wegscheider K, Kersten JF, Icks A, Mühlhauser I Increased use of hip protectors in nursing homes: economic analysis of a cluster randomized, controlled trial. J Am Geriatr Soc.

